# Interchromosomal Transfer of Immune Regulation During Infection of Barley with the Powdery Mildew Pathogen

**DOI:** 10.1534/g3.117.300125

**Published:** 2017-08-08

**Authors:** Priyanka Surana, Ruo Xu, Gregory Fuerst, Antony V. E. Chapman, Dan Nettleton, Roger P. Wise

**Affiliations:** * Interdepartmental Bioinformatics and Computational Biology Program, Iowa State University, Ames, Iowa 50011; † Department of Plant Pathology and Microbiology, Iowa State University, Ames, Iowa 50011; ‡ Department of Statistics, Iowa State University, Ames, Iowa 50011; § Corn Insects and Crop Genetics Research, United States Department of Agriculture-Agricultural Research Service, Iowa State University, Ames, Iowa 50011; ** Interdepartmental Genetics and Genomics, Iowa State University, Ames, Iowa 50011

**Keywords:** immune regulation, expression quantitative trait locus (eQTL), barley (*Hordeum vulgare*), *Blumeria graminis*, host–pathogen interactions

## Abstract

Powdery mildew pathogens colonize over 9500 plant species, causing critical yield loss. The Ascomycete fungus, *Blumeria graminis* f. sp. *hordei* (*Bgh*), causes powdery mildew disease in barley (*Hordeum vulgare* L.). Successful infection begins with penetration of host epidermal cells, culminating in haustorial feeding structures, facilitating delivery of fungal effectors to the plant and exchange of nutrients from host to pathogen. We used expression Quantitative Trait Locus (eQTL) analysis to dissect the temporal control of immunity-associated gene expression in a doubled haploid barley population challenged with *Bgh*. Two highly significant regions possessing *trans* eQTL were identified near the telomeric ends of chromosomes (Chr) 2HL and 1HS. Within these regions reside diverse resistance loci derived from barley landrace *H. laevigatum* (*MlLa)* and *H. vulgare* cv. Algerian (*Mla1)*, which associate with the altered expression of 961 and 3296 genes during fungal penetration of the host and haustorial development, respectively. Regulatory control of transcript levels for 299 of the 961 genes is reprioritized from *MlLa* on 2HL to *Mla1* on 1HS as infection progresses, with 292 of the 299 alternating the allele responsible for higher expression, including Adaptin Protein-2 subunit μ AP2M and Vesicle Associated Membrane Protein VAMP72 subfamily members VAMP721/722. AP2M mediates effector-triggered immunity (ETI) via endocytosis of plasma membrane receptor components. VAMP721/722 and SNAP33 form a Soluble N-ethylmaleimide-sensitive factor Attachment Protein REceptor (SNARE) complex with SYP121 (PEN1), which is engaged in pathogen associated molecular pattern (PAMP)-triggered immunity via exocytosis. We postulate that genes regulated by alternate chromosomal positions are repurposed as part of a conserved immune complex to respond to different pathogen attack scenarios.

Powdery mildew fungi infect >9500 plant species, resulting in more yield loss than any other single type of plant disease (Inuma *et al.* 2007). Cereal grains, particularly wheat and barley, are among the most important agricultural crops that suffer. Barley powdery mildew, caused by the filamentous Ascomycete fungus, *Blumeria graminis* f. sp. *hordei* (*Bgh*), has been developed into a model system to study the relationship between obligate biotrophs and their host plants. Infection of barley by *Bgh* begins when a conidiospore lands on the leaf surface, germinates, and differentiates into an appressorial germ tube. This is followed by penetration of the epidermal cells and development of haustoria, which facilitate delivery of pathogen effectors to the host plant and uptake of nutrients from the plant to the fungus.

To defend themselves from invading pathogens, such as *Bgh*, plants have evolved innate and induced immune responses. Innate immunity is species nonspecific and recognizes conserved PAMPs via pattern recognition receptors at the cell surface; this initiates intracellular signaling pathways that lead to PAMP-triggered immunity (PTI) (Macho and Zipfel 2014). Interconnected with PTI is a second layer, designated ETI (Cui *et al.* 2015). ETI is considered species-specific and is activated when a plant resistance protein interacts, directly or indirectly, with corresponding pathogen-secreted effector proteins, which alter host processes to promote nutrient acquisition and colonization (Bent and Mackey 2007; Jacob *et al.* 2013; Cesari *et al.* 2014). This interaction triggers defense signaling in the cytoplasm or the nucleus, resulting in programmed cell death (Li *et al.* 2015).

Reaction to *Bgh* is controlled by several loci distributed across the barley genome, termed *Ml* (for Mildew resistance locus). Positioned on the short arm of Chr 1H (Wei *et al.* 2002), the *Mla* locus harbors over 30 alleles that confer varying levels of resistance when corresponding avirulence (AVR) effectors are present in the pathogen (Shen *et al.* 2003; Halterman and Wise 2004; Seeholzer *et al.* 2010). *Mla* alleles encode coiled-coil, nucleotide binding, leucine-rich repeat (NLR) proteins that accumulate in the nucleus after recognition of corresponding AVR effector proteins from *Bgh* (Li *et al.* 2015). After recognition, MLA dissociates the MYB6 transcriptional activator from the WRKY1 repressor and promotes its binding to corresponding *cis*-regulators, initiating disease resistance signaling (Chang *et al.* 2013).

Despite the observation that plant resistance to disease is often associated with single segregating loci, the underlying genetic architecture that contributes to the ultimate phenotype can be comprised of hundreds to thousands of genes (Kliebenstein 2009a). eQTL analysis is used to associate gene expression measurements with polymorphisms in a segregating population (Farrall 2004; Gilad *et al.* 2008). eQTLs are classified as *cis*- or *trans*-acting by comparing their genomic location to the position of the target genes. A *cis*-regulatory change is inferred when the eQTL coincides with the target gene, whereas a *trans*-regulatory change is assumed when the eQTL is not near the target gene (Wittkopp 2005). Regions of the genome that associate with the expression of many genes are referred to as eQTL hotspots (Chesler *et al.* 2005; Hansen *et al.* 2008), which tend to act in *trans*, regulating >1000 genes in some cases (West *et al.* 2007; Hansen *et al.* 2008; Li *et al.* 2013). Thus, eQTL hotspots can be used to characterize chromosomal positions of major regulators (de Koning and Haley 2005), including, for example, those involved in disease resistance (Mozhui *et al.* 2008; Kliebenstein 2009b; Chen *et al.* 2010; Moscou *et al.* 2011b; Samad-Zamini *et al.* 2017).

We sought to address the temporal regulation of immunity in barley–powdery mildew interactions by eQTL analysis. Two main questions were addressed: (i) which genes are regulated by *Ml* loci, and (ii) how are these genes regulated. Briefly, genome-wide transcriptome analysis of the barley Q21861 × SM89010 doubled-haploid population identified two major regions containing *trans* eQTL near the telomeric ends of Chr 2HL and 1HS. Residing within these regions are the resistance loci *MlLa* (connected to intermediate quantitative resistance) and *Mla* (conferring strong qualitative resistance), which associate with expression levels of 961 and 3296 genes, respectively. Intriguingly, the *MlLa* region controlled expression during *Bgh* penetration while the *Mla* region controlled expression during development of fungal haustoria. Moreover, of the 961 genes under transcriptional control of the *MlLa* locus, 299 are reallocated to *Mla* as infection progressed. These findings suggest that a conserved immune regulon is activated by disparate resistance loci to achieve immunity in response to multiple infection stages.

## Materials and Methods

### Plant growth, RNA extraction, and GeneChip hybridization

The 75 Q21861 × SM89010 (QSM) doubled haploid barley lines (Steffenson *et al.* 1995) were derived from a single F_1_ plant, and have been maintained via single seed descent (Moscou *et al.* 2011b). Seedlings were grown in two 98-cone-tainer trays in a climate-controlled greenhouse using a randomized block design. Each tray contained five seedlings/cone of the 75 QSM lines plus four replicates of each parent. Seven days after sowing, both trays were inoculated with *Bgh* isolate 5874 (*AVR_a1_*, *AVR_a6_*, *vir_a8_*, *vir_a13_*, and *AVR_La_*) at an average density of 200 conidiospores per cm^2^. The five seedlings were harvested, pooled, and placed in liquid nitrogen for each progeny and parent line at 16 and 32 hr after inoculation (HAI). Total RNA was isolated using a hot (60°) phenol/guanidine thiocyanate method (Caldo *et al.* 2004). Labeling, hybridization, washing, and scanning were performed according to standard Affymetrix protocols at the Iowa State University GeneChip Core facility using 22K Barley1 GeneChip expression arrays (Affymetrix part number 900515) (Close *et al.* 2004).

### Phenotyping

*Bgh* isolate 5874 was propagated on *Hordeum* *vulgare* cv. Morex at 18° while in 16 hr of light and 8 hr of darkness. *H. laevigatum*-derived lines were obtained from the USDA-Germplasm Resources Information Network (GRIN). Alleles at the *Mla* locus were amplified via polymerase chain reaction (PCR) using primers Mla_LRR F and R (Supplemental Material, Table S1), which amplified the LRR region, spanning ∼850 nucleotides between positions 1897 and 2725 bp relative to the *Mla6* genomic sequence (AJ302293.1). Sequence verification was done by alignment against known *Mla* alleles (Seeholzer *et al.* 2010), and the alleles in Q21861 and SM89010 were assigned as *Mla1* (AY009939.1) and *Mla8* (GU245940.1) respectively.

To phenotype the QSM population, five 98-cone trays of Q21861, SM89010, and 75 QSM progeny were planted in sterilized potting soil. Each of the five experimental trays (7 rows × 14 columns) consisted of five seedlings for each parent (Q21861 and SM89010) and one seedling for each QSM progeny. One seedling was planted per cone and genotypes were completely randomized within each of the five trays. Seedlings were grown in a 20° Controlled glasshouse to 10 cm prior to the application of treatment. Seedlings were inoculated with fresh *Bgh* conidiospores and trays were transferred to a growth chamber at 18° (16 hr light and 8 hr darkness) where the trays were stored side-by-side in randomly assigned positions. Six d after inoculation, all leaves were photographed and scored for infection type (Table S2). The entire procedure was repeated three times in successive weeks.

### Expression QTL mapping

eQTL analysis was carried out separately for the 16 and 32 HAI time points. Each of 22,840 probe sets was scanned for association with each of 377 nonredundant markers (Moscou *et al.* 2011b) using data from the 75 QSM lines. For probe set *i* = 1, …, 22,840 and marker *j* = 1, …, 377, a simple linear regression model was fitted to 75 data points with robust multi-array average (RMA)-normalized expression level as the response and an indicator of marker genotype as the explanatory variable. For missing genotypes, observed flanking marker genotypes were used with linear interpolation to impute a continuous genotype value in the interval [0,1]. A t-statistic (t*_ij_*) for testing the significance of the slope coefficient in the simple linear regression model was computed to quantify the strength of association between probe set *i* and marker *j*. For each probe set *i*, T*_i_* = the maximum value of |t*_ij_*| over markers *j* = 1, …, 377 was computed, and the identity of the marker yielding the maximum value was recorded.

To identify statistically significant associations of expression level with marker genotype, the statistics T_1_, …, T_22,840_ were first converted to p-values (p_1_, …, p_22,840_) using a standard permutation approach that accounts for multiple testing across the 377 markers (Churchill and Doerge 1994). Specifically, the values of T_1_, …, T_22,840_ were recomputed for each value of 100 permutations, where for each permutation, the 75 expression vectors associated with the 75 QSM lines were randomly assigned to the 75 genotype vectors associated with the 75 QSM lines. This yielded a null distribution of 22,840 × 100 permutation test statistics. The p-value for the original statistic T*_i_* was given by the proportion of the 22,840 × 100 permutation test statistics ≥ T*_i_*. To account for multiple testing across 22,840 probe sets, the 22,840 permutation p-values were converted to q-values (q_1_, …, q_22,840_) (Benjamini and Hochberg 1995). To control false discovery rate (FDR) at level 0.001, the association between probe set *i* and its most strongly associated marker was declared significant if and only if its q-value (q*_i_*) was ≤0.001.

For this experiment, we interrogated the data set for only the most associated markers to identify the primary eQTL that had the greatest impact on gene expression. While other secondary and tertiary eQTL can also exert influence, we focused on changes in the most associated regulatory eQTL, with the hypothesis that it represents the most significant modification in the underlying biology of the gene in question.

### Differential expression analyses for QSM progeny groups

The 75 QSM progeny were grouped by the four *Ml* gene + allele combinations—(1) *Mla1*, *MlLa*; (2) *Mla1*, *mlLa*; (3) *mla1*, *MlLa*; and (4) *mla1*, *mlLa*—and analyzed using R package limma in Bioconductor (Ritchie *et al.* 2015). A model was fitted with expression value as response and treatment (genotype * time point) as explanatory variables. p-values were adjusted for multiple testing using R package q-value (Storey *et al.* 2015). Genes that had a q-value of ≤0.01 were considered significant.

### Comparing QSM progeny lines to Q and SM parents

The gene expression for each of the QSM progeny was compared to the gene-wise median for Q and SM parents combined for the respective time point. A log-fold difference of 2 between QSM progeny and the parents was considered a sign of differential expression.

### Transcript pattern analyses of published data sets

We conducted linear model analyses of the normalized signal intensities for each of the 22,840 Barley1 probe sets (Close *et al.* 2004) using packages in BioConductor/R (Gentleman *et al.* 2004; R Core Team 2016). RMA normalization and data transformation was done using package affy (Gautier *et al.* 2004). Linear model analysis and multiple testing adjustments were conducted using package limma (Ritchie *et al.* 2015) and q-value (Storey *et al.* 2015). The model used RMA normalized expression values as the response, and treatment (genotype * time point) as the input variable. Contrasts were used to compare transcript levels between treatments after infection with *Bgh* 5874. Q-values were estimated using the smoother method described in Storey and Tibshirani (2003).

Custom R program scripts were used to parse for highly similar patterns of expression among incompatible and compatible interactions up to 16 HAI, followed by significant (q-value ≤ 0.01) divergence from 16 to 32 HAI. This was used to distinguish two opposing scenarios: (i) downregulation of the transcript in the compatible interaction after 16 hr, as compared to paired time points in the incompatible interaction (designated “PacMan”), or (ii) upregulation of the transcript in the compatible interaction after 16 hr, as compared to paired time points in the incompatible interaction (designated “iPacMan”).

### MlLa marker design and testing

The two markers, MLOC_20004 and MLOC_25435 on Chr 2H, represent the outermost boundaries of a region flanked by recombination breakpoints and implicated to contain *MlLa*, at 621 and 624.5 Mb, respectively. Using the available barley Morex genome on Ensembl (Assembly 082214v1, INSDC Assembly GCA_000326085.1) (International Barley Genome Sequencing Consortium *et al.* 2012), 29 high-confidence candidates were selected. To further delineate the region that contains *MlLa*, markers were generated to identify genes within this region that cosegregated with the *MlLa* phenotype. Multiple primer sets were used to amplify candidate genes using standard *Taq* polymerase (Invitrogen, Carlsbad, CA) from cultivars SM89010 (*Mla8* and *MlLa*) and Q21861 (*Mla1* and *mlLa*). Any primer sets that showed potential as PCR-based markers (presence/absence or band-shift) were utilized on the QSM DH population (Table S3) and *H. laevigatum*-derived lines (Table S4). Those that showed no discernible difference between the two parents were sequenced to design cleaved amplified polymorphic sequence markers.

### Computational prediction of secretion-associated genes/proteins

To reliably map the *Arabidopsis* proteome to the Barley1 GeneChip probe sets, we obtained empirical scores for coverage and identity. Rice (*Oryza sativa* subsp. *japonica*) was selected as the monocot representative as it had the highest number of protein sequences (3716) in Swiss-Prot (UniProt Consortium 2015) as compared to barley (350 sequences). The coverage and identity scores were based on a Rice–*Arabidopsis* comparison of protein sequences obtained from Swiss-Prot using blastp in the BLAST+ command line suite (Camacho *et al.* 2009). This alignment gave an average of 86% coverage and 60% identity among the orthologous sequence pairs.

Next, proteinsequences for *Arabidopsis* assembly Araport11 were obtained from TAIR (www.arabidopsis.org) and compared against the Harvest 21 assembly that was used to derive Barley1 GeneChip probe sets (Close *et al.* 2004) using blastx in the BLAST+ command line suite (Camacho *et al.* 2009). Coverage was calculated using the query as well as the subject and the maximum of the two was used. Sequences that aligned with 86% coverage and 60% identity were selected as candidates for homologs.

### Connecting alternatively regulated genes to biological function

Of the 344 membrane trafficking encoding genes associated with 2HL at 16 HAI and/or 1HS at 32 HAI (Table S5), we focused on the 37 Barley1 probe sets that exhibit alternate regulation between the two developmental stages. *Arabidopsis* proteins corresponding to these probe sets were interrogated for previously established protein–protein interactions in BioGRID (Stark *et al.* 2006). This resulted in a network of 129 nodes partitioned into 19 communities developed using the igraph package (Csardi and Nepusz 2006) in R (R Core Team 2016). These communities had a high modularity of 0.842, which reflects dense connections within communities and sparse connections across communities.

### Data availability

All MIAME-compliant GeneChip profiling data from the QSM eQTL experiment are available as accession BB96 at the PLEXdb expression resource for plants and plant pathogens, as well as accession GSE68963 at NCBI-GEO. The previously published PLEXdb data set, BB4, is also available as NCBI-GEO accession GSE33396.

Table S1 details the PCR primers used in this study. Table S2 shows infection type data for three replications. Table S3 contains the complete QSM genetic map. Table S4 details the genotyping and phenotyping results for *H. laevigatum* and *H*. *laevigatum*-derived lines. Table S5 features genes associated with membrane trafficking. Table S6 lists genes associated with each marker on the QSM map. Table S7 provides differential expression results for comparison between CI 16151 and CI 16155 (BB4), and between QSM progeny groups (*Mla1* and *mlLa*) and (*mla1* and *mlLa*). Table S8 shows the modes of alternate regulation for the genes transferred between 2HL at 16 HAI and 1HS at 32 HAI. Figure S1 shows a subnetwork connected via PEN1 (SYP121) in community A and VAMP721, SNAP33, and KEULE in community B. Figure S2 illustrates a community with the alternatively regulated AP2M as the hub that interacts with the remaining five proteins, including AP2A1.

## Results

### Mla1 is epistatic to MlLa

**Figure 1:**
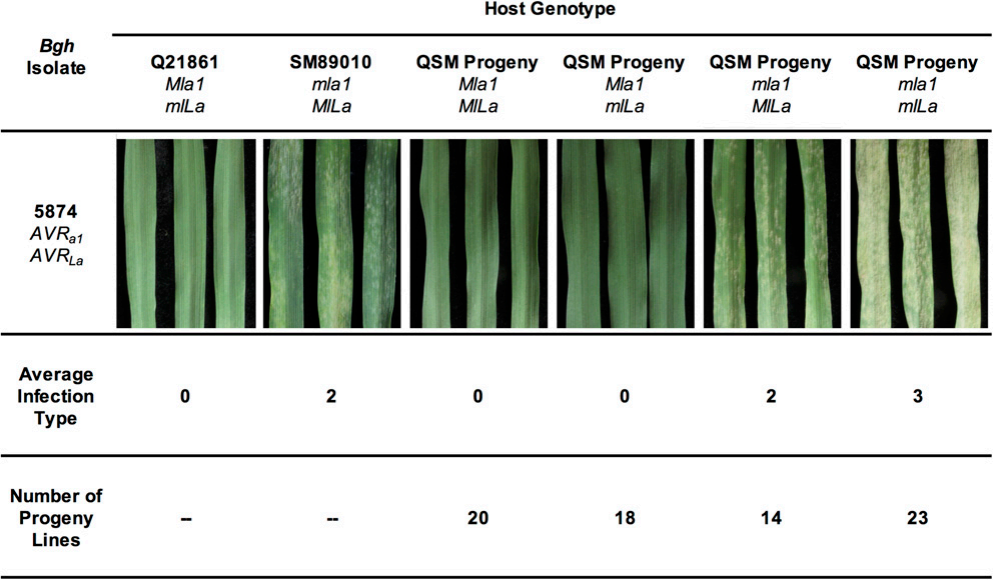
Infection types of Q21861, SM89010, and their recombinant progeny containing different combinations of *Ml* genes. Seven-day-old seedlings of each genotype were inoculated with *Bgh* isolate 5874 and photographed 6 d later. The three leaves shown represent an average infected plant for each genotypic group. An average infection phenotype of <2.5 across three replications was considered resistant (incompatible interaction), whereas a value of ≥2.5 was considered susceptible (compatible interaction). The values are rounded to the nearest 0.5. QSM, Q21861 × SM89010.

In order to determine if interactions between *Mla* and *MlLa* had an effect on infection phenotype (IT), the 75 QSM barley lines (Steffenson *et al.* 1995) were inoculated with *Bgh* isolate 5874. This isolate harbors both the *AVR_a1_* and *AVR_La_* AVR effectors, and thus, was used to monitor the various infection types segregating in the QSM progeny. Figure 1 illustrates that Q21861 confers an IT of 0 in response to *Bgh* 5874, whereas SM89010 imparts an average IT of 2 with the same isolate. A 1259 cM transcript-derived marker map of the QSM population (Moscou *et al.* 2011b) was used to position *Mla1* and *MlLa*. This map is divided into 377 bins across seven Chr and harbors 1494 Barley1 GeneChip-derived markers (Table S3).

Infection phenotyping (Wei *et al.* 1999) positioned a *Bgh* resistance specificity at the *Mla* locus on Chr 1HS (1H.05), and another on the distal end of Chr 2HL. The second resistance that mapped to 2H.67 colocalized with the position associated with *MlLa* (Schweizer and Stein 2011). ITs for three replications of each QSM recombinant line are detailed in Table S2.

*MlLa* is derived from the barley landrace *H. laevigatum*, but its precise position and sequence are currently unknown. Therefore, to determine whether we were indeed tracking the *MlLa* locus, we compared infection types and *MlLa* flanking marker sequences of SM89010 (the QSM parent line putatively harboring *MlLa*) to *H. laevigatum* and 10 other *H. laevigatum*-derived accessions (Dros 1957). SM89010 displayed identical infection type and DNA marker sequences to *H. laevigatum*, as well as the *H. laevigatum*-derived Minerva and Vada. This suggests that the locus mapped is likely *MlLa* (Table S4). *Mla*-derived sequences were amplified and aligned against known *Mla* alleles (Seeholzer *et al.* 2010), confirming that Q21861 possessed *Mla1*, which was consistent with the observed IT of 0 associated with this allele.

Taking epistatic interactions into account, it was conceivable that some of the recombinant progeny in the QSM population would display an IT of 0 in response to *Mla1*, but still contain *MlLa*. Indeed, all 38 lines that contained *Mla1* displayed an IT of 0, whereas the 14 lines that contained *MlLa* only displayed an average IT of 2, and lines that contained neither *Mla1* nor *MlLa* exhibited an average IT of 3. Genotypes for the double haploid progeny fit a ratio of 1:1:1:1 (*χ*^2^ 1:1:1:1 = 2.28; p-value = 0.5164) and a phenotypic ratio of 2 ^(IT = 0)^:1 ^(IT = 2)^:1^(IT = 3)^ (*χ*^2^ 2:1:1 = 2.17; p-value = 0.3373) as expected for an epistatic interaction.

### Trans eQTL hotspots are associated with MlLa during Bgh penetration and Mla during haustorial development

The QSM population is ideal for identifying regulatory components that reprogram the defense transcriptome of barley in response to inoculation with *Bgh*. Parental and QSM progeny lines were inoculated with *Bgh* isolate 5874 and harvested at 16 and 32 HAI; time points that are associated with appressorial penetration and early formation of haustoria, respectively (Caldo *et al.* 2004, 2006; Moscou *et al.* 2011a). Gene expression for each of the 166 experimental units was estimated using the 22K Barley1 GeneChip (Close *et al.* 2004) (BB96 in PLEXdb and GSE68963 in NCBI-GEO).

A simple linear regression model was fit to 75 data points with normalized expression level as the response and an indicator of marker genotype as the explanatory variable to identify eQTLs. Significance thresholds were determined by using q-values to control the FDR (Benjamini and Hochberg 1995). Table S6 lists all 22,840 probe sets along with their most associated eQTL marker and its level of significance at 16 and 32 HAI. With the FDR controlled at 0.001, 357 eQTL marker positions (bins) were classified as most significantly associated with at least one probe set (gene) at 16 HAI; five of these eQTL markers were of highest significance to at least 100 probe sets each. For the same threshold, 371 positions were classified as the top association for at least one probe set at 32 HAI. Likewise, 6 of these 371 were found to be most associated with at least 100 probe sets. In simulations performed with a multinomial distribution, the probability for an eQTL marker position to be randomly associated with 100 probe sets by chance is essentially 0 with eQTL marker associations controlled at q-value ≤ 0.001. Thus, for the purposes of this manuscript, only loci associated with > 100 probe sets were considered eQTL hotspots.

**Figure 2:**
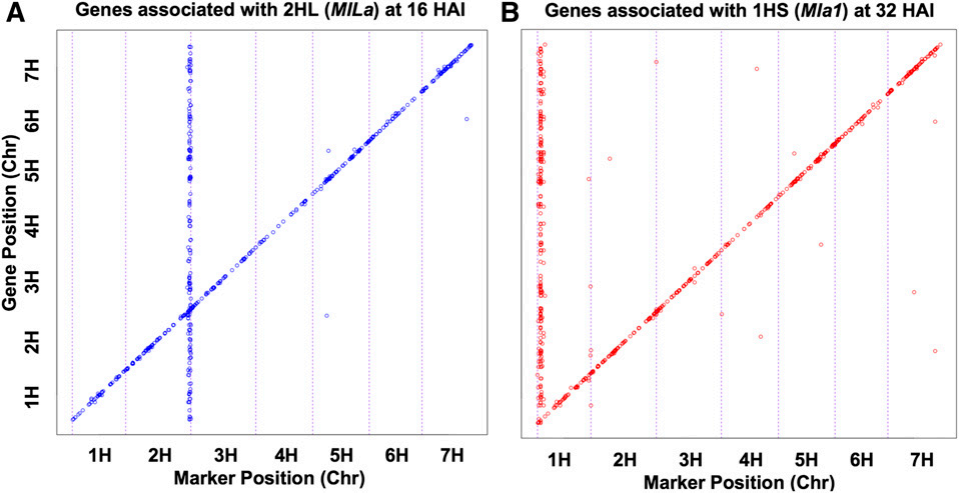
*Trans* eQTL hotspots localize to chromosomes 2HL and 1HS. Of the 1494 probe sets that are positioned on the QSM genetic map, (A) illustrates significant off-diagonal probe sets associated with Chr 2HL at 16 HAI with *Bgh* 5874 (q-value ≤ 0.001), and (B) demonstrates significant off-diagonal probe sets associated with 1HS at 32 HAI. These off-diagonal probe sets on 2HL and 1HS are indicative of *trans* eQTL hotspots. Chr, chromosome; eQTL, expression Quantitative Trait Locus; HAI, hr after inoculation; QSM, Q21861 × SM89010.

The five eQTL hotspots significant at 16 HAI, corresponding to penetration of epidermal cells by *Bgh*, map to linked positions on Chr 2HL Bins 63 and 65–68 (*MlLa* region) and associate with a total of 3103 genes (Table S6). Likewise, the six hotspots significant at 32 HAI, corresponding to formation of *Bgh* haustorial feeding structures and delivery of secreted effectors into the host, localized to Chr 1HS Bins 3–8 (including *Mla1*) and associate with 5070 genes. In this report, 2HL will be the designation for Chr 2H Bins 63 and 65–68 and 1HS refers to Chr 1H Bins 3–8. Figure 2 illustrates off-diagonal probe sets, indicative of *trans* eQTL, including those associated with 2HL at 16 HAI and 1HS at 32 HAI.

**Figure 3:**
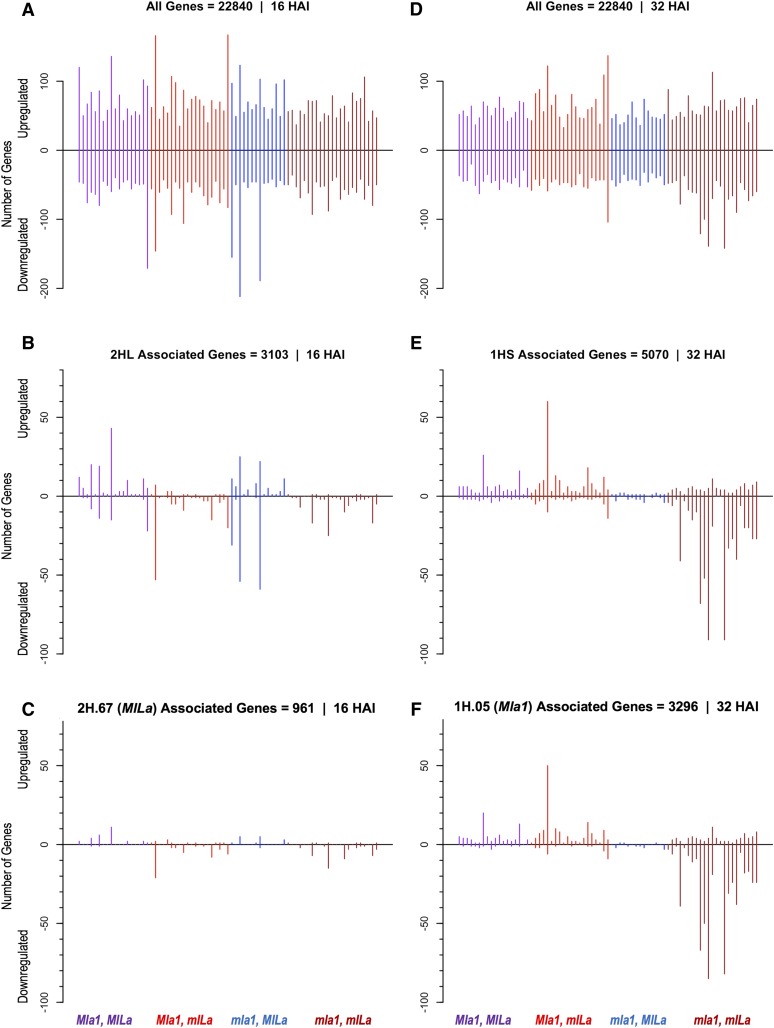
Number of genes up- and downregulated in each QSM progeny for 16 and 32 HAI as compared to the gene-wise median for the Q and SM parent lines. At 16 HAI, (A) all genes, (B) genes associated with 2HL, and (C) genes associated with 2H.67 (*MlLa*) are plotted for each QSM progeny. At 32 HAI, (D) all genes, (E) genes associated with 1HS, and (F) genes associated with 1H.05 (*Mla1*) are represented. The purple, red, blue and brown lines refer to QSM progeny genotype (*Mla1* and *MlLa*), (*Mla1*, and *mlLa*), (*mla1* and *MlLa*), and (*mla1* and *mlLa*), respectively. Within each group, the barley lines are in numerical order (Table S2). Note that the graphs are plotted on different scales. HAI, hr after inoculation; QSM, Q21861 × SM89010.

We then wished to examine any changes within each of the four genotypic progeny groups and identify the alleles at 2HL and 1HS eQTL that suppress or upregulate associated genes. To do this, we compared the gene expression for each progeny line against the median of the parents. Figure 3 illustrates the number of up- and downregulated genes in each QSM line ordered by genotype. In Figure 3, B and C at 16 HAI, there is an overrepresentation of upregulated genes in QSM lines that possess the functional *MlLa* gene (SM genotype), *i.e.*, (*Mla1* and *MlLa*; purple) and (*mla1* and *MlLa*; blue), whereas there is a clear trend of downregulated genes in QSM progeny lacking the *MlLa* gene (red and brown). Similarly, in Figure 3, E and F at 32 HAI, there is an overrepresentation of upregulated genes in QSM lines with the functional *Mla* gene (Q genotype), *i.e.*, (*Mla1* and *MlLa*; purple) and (*Mla1* and *mlLa*; red), whereas there is a clear trend of downregulated genes in QSM progeny lacking either *R* gene (brown). This indicates that the *MlLa* and *Mla1* alleles in the two eQTL regions tend to be associated with upregulation of associated genes rather than suppression.

### Transcript pattern analysis associates eQTL hotspots on Chr 2HL and 1HS with compatibility or incompatibility

Next, we integrated the current eQTL results with previous Barley1 GeneChip expression data to examine changes in transcript accumulation across developmental time. Accession BB4 (PLEXdb; GSE33396 in NCBI-GEO) facilitated comparison with independent incompatible and compatible interactions mediated by the *Mla1* and *Mla13* alleles, respectively, at six different time points (0, 8, 16, 20, 24, and 32 HAI) with *Bgh* isolate 5874 (*AVR_a1_*, *AVR_La_*, and *avr_a13_*) (Caldo *et al.* 2004).

**Figure 4:**
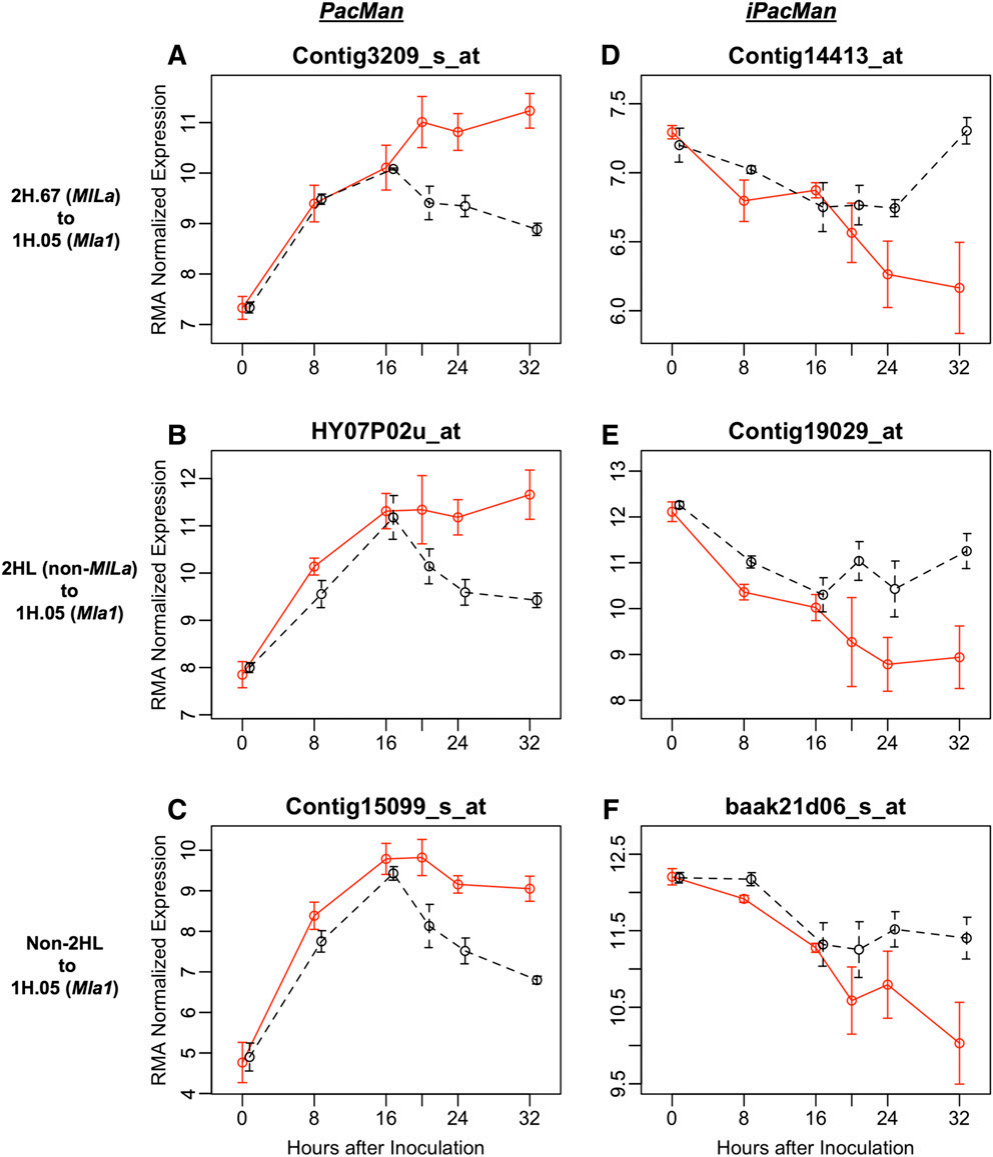
Examples of genes exhibiting PacMan or iPacMan patterns of transcript accumulation. The red and black lines represent RMA-normalized expression for barley lines CI 16137 (*Mla1*) and CI 16155 (*Mla13*) infected with *Bgh* isolate 5874 (*AVR_a1_*, *AVR_a6_*, and *avr_a13_*), respectively. The following genes are associated with various eQTL at 16 HAI, and then are repurposed to associate with 1H.05 (*Mla1*) at 32 HAI. (A) Contig3209_s_at (blue copper-binding protein homolog) from 2H.67 (*MlLa*). (B) HY07P02u_at (anthranilate synthase α 2 subunit) from non-*MlLa* Chr 2HL hotspot (2H.66). (C) Contig15099_s_at (pathogenesis-related protein 4) from non-2HL hotspot (3H.57). (D) Contig14413_at (auxilin-like protein) from 2H.67 (*MlLa*). (E) Contig19029_at (bacterial-induced peroxidase precursor) from non-*MlLa* Chr 2HL hotspot (2H.66). (F) baak21d06_s_at (Unknown) from non-2HL hotspot (7H.03). Chr, chromosome; eQTL, expression Quantitative Trait Locus; HAI, hr after inoculation; iPacMan, genes upregulated from 16 to 32 HAI during compatible interactions, as compared to the equivalent time points in incompatible interactions; PacMan, genes downregulated during compatible interactions after 16 HAI, as compared to corresponding time points in incompatible interactions.

Of particular interest were two classes of transcript accumulation; both were highly similar among incompatible and compatible interactions up to 16 HAI, coinciding with germination of *Bgh* conidiospores and formation of appressoria, followed by significant divergence from 16 to 32 HAI, during development of the perihaustorial membrane between fungal haustoria and host epidermal cells (Caldo *et al.* 2004). Computational pattern analysis was used to distinguish the two opposing scenarios: The first represents genes downregulated during compatible interactions after 16 HAI, as compared to corresponding time points in incompatible interactions; this pattern was designated PacMan (Figure 4, A–C and Table 1). In contrast, the second represents genes upregulated from 16 to 32 HAI during compatible interactions, as compared to the equivalent time points in incompatible interactions; this pattern was designated iPacMan (Figure 4, D–F and Table 1). The first scenario may represent positive regulators of host immune signaling that are suppressed by the pathogen during compatible interactions, whereas the second may represent a situation where the pathogen is inducing host factors for disease susceptibility. Of the 3103 genes associated with the eQTL hotspot cluster on Chr 2HL, 321 and 258 exhibited PacMan and iPacMan expression patterns, respectively. Similarly, of the 5070 genes associated with 1HS, 404 are associated with a PacMan pattern, whereas 606 displayed an iPacMan pattern (Table 1 and Table S6).

**Table 1:** Number of genes associated with each hotspot in the Chr 2HL and 1HS clusters and the number represented by PacMan and iPacMan expression patterns, respectively

Position (Chr.Bin)	Associations*^a^*	Expression Pattern*^b^*	
		PacMan	iPacMan
2H.63	217	17	10
2H.64*^c^*	26	0	0
2H.65	261	33	24
2H.66	1331	163	167
2H.67 (*MlLa*)	961	100	26
2H.68	333	8	31
2HL total	3103	321	258
1H.03	261	13	12
1H.04	276	14	6
1H.05 (*Mla1*)	3296	341	431
1H.06	783	21	121
1H.07	114	7	13
1H.08	340	8	23
1HS total	5070	404	606

Chr, chromosome; PacMan, genes downregulated during compatible interactions after 16 HAI, as compared to corresponding time points in incompatible interactions; iPacMan, genes upregulated from 16 to 32 HAI during compatible interactions, as compared to the equivalent time points in incompatible interactions.

a Associated genes are represented at 16 HAI for Chr 2H and 32 HAI for Chr 1H hotspots at a q-value ≤ 0.001. The annotations for these genes are presented in Table S6.

b Expression pattern changes across developmental time in the comparison of incompatible (*Mla1-AVR_a1_*) and compatible (*Mla13-avr_a13_*) barley-*Bgh* interactions (Accession BB4 at PLEXdb.org). Described further in *Pattern Analyses of Published Data Sets* under *Materials and Methods*.

c Marker bin not included in analyses as the number of associations is below 100 but shown here for informational purposes.

The genotypes represented by PLEXdb accession BB4 lack *MlLa*, but do possess *Mla1*. Therefore, when inoculated with *Bgh* 5874 (*AVR_a1_*, *AVR_La_*, and *avr_a13_*), a comparison of differential gene expression can be made between QSM progeny groups (*Mla1* and *mlLa*; resistant) and (*mla1* and *mlLa*; susceptible) in BB96, and between CI 16151 (*Mla1* and *mlLa*; resistant) and CI 16155 (*Mla13* and *mlLa*; susceptible) from BB4. At 32 HAI, of the 1712 genes that are differentially expressed between CI 16151 and CI 16155, 1356 (∼80%) are also differentially expressed between (*Mla1* and *mlLa*) and (*mla1* and *mlLa*) in BB96. Of these 1356 genes, 559 are upregulated and 797 are downregulated in the *Mla1* and *mlLa* QSM progeny group (Table S7).

### Temporal regulation of immunity

Up to now, a handful of genes (and their encoded proteins) have been functionally connected to NLR-mediated pathways (Shirasu 2009; Jacob *et al.* 2013), but these interactions have not been associated with specific stages of pathogen development. Previous studies have shown that MLA1 interacts with SGT1 (suppressor of the G2 allele of *skp1*) (Bieri *et al.* 2004), which binds SKP1, a component of the Skp1-Cullin-F-box ubiquitin ligase complex, and facilitates regulation of defense in both yeast and plants (Kitagawa *et al.* 1999; Azevedo *et al.* 2002). Along with RAR1 (required for *Mla12* resistance 1) and HSP90 (Heat Shock Protein 90), SGT1 positively controls the steady state levels of resistance proteins recognizing viral, bacterial, oomycete, or fungal pathogens (Azevedo *et al.* 2002; Takahashi *et al.* 2003; Schulze-Lefert 2004; Shirasu 2009). Our findings show that SGT1 is associated with 2H.67 (*MlLa* locus) at 16 HAI, whereas HSP90 is associated with 1H.05 (*Mla1*) at 32 HAI. However, RAR1 does not associate significantly with either 2HL or 1HS, or any other eQTL marker.

Formerly, HvWRKY10, HvWRKY19, and HvWRKY28 were shown to positively regulate *Mla*-triggered immunity to *Bgh*; however, current evidence suggests that these three nuclear-localized WRKY transcription factors do not directly bind MLA (Meng and Wise 2012). Of these, we show that WRKY19 is regulated (either directly or indirectly) by 2H.67 (*MlLa*) at 16 HAI, whereas WRKY10 is associated with 1H.05 (*Mla1*) at 32 HAI. Additional WRKYs associated with 2HL at 16 HAI include WRKY20, WRKY33, WRKY34, WRKY41, WRKY46, and WRKY61. From this group, WRKY33, WRKY41, and WRKY46 change association from 2H.65, 2H.66, and 2H.67 (*MlLa*) at 16 HAI, respectively, to 1H.05 (*Mla1*) at 32 HAI (Table S6).

Lastly, the previously known defense-related proteins, syntaxin (VAMP727), thaumatin, and SOD1 display no significant associations at 16 HAI, but were associated with 1H.08, 1H.05 (*Mla1*), and 1H.06 at 32 HAI, respectively. BLN1, a negative regulator of basal defense (Meng *et al.* 2009; Xu *et al.* 2015), was associated with 2H.68 during penetration, but 1H.07 during haustorial growth, implying control by unlinked host loci during pathogen development.

To further extend the temporal analysis of the known NLR-associated proteins above, we also queried the 2HL and 1HS associations for encoded proteins annotated to membrane trafficking, including the secretory pathway, nuclear and vesicle transport, signaling G-proteins, and their known interactors (Stark *et al.* 2006; Paul *et al.* 2014; Rutter and Innes 2017). This identified 356 distinctive probe sets encoding several SNAREs, Rab GTPases (part of the Ras superfamily of small GTPases), tethering factors, COat Protein complex (COPs), and Clathrin-Coated Vesicle proteins (CCVs). Of these 356 probe sets, 80 were associated with 2HL at 16 HAI, 126 associated with 1HS at 32 HAI, and 38 were associated with both 2HL at 16 HAI and then 1HS at 32 HAI (Table 1 and Table S6). Taken together, the current eQTL analysis identifies proteins coupled to NLR-mediated signaling, but also provides new associations that appear to play important temporal roles during penetration, haustorial growth, or both.

### Alternating temporal control: prioritizing immune regulation from 2HL (associated with penetration) to 1HS (associated with haustorial development)

**Figure 5:**
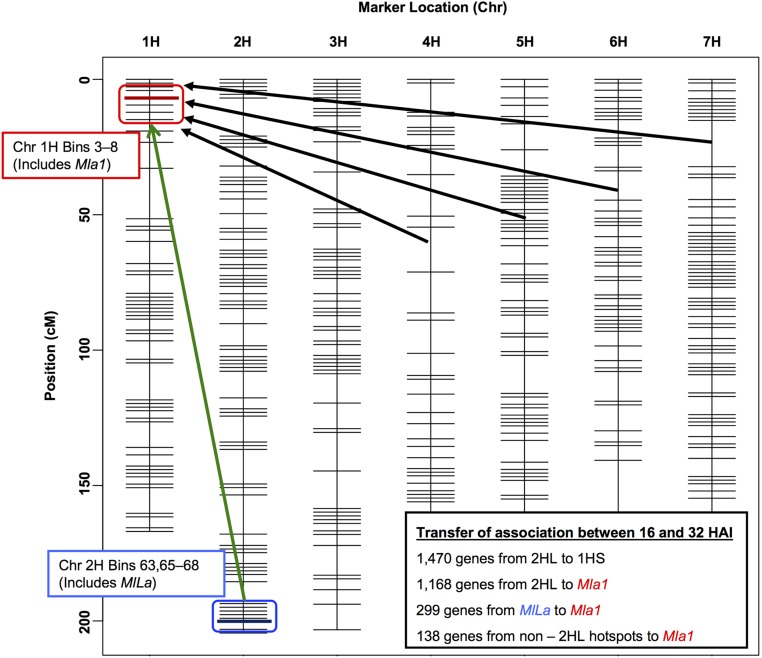
Illustration of 16 and 32 HAI eQTL hotspots projected onto the QSM genetic map. Chr 1HS (*Mla1* region, red box) and 2HL (*MlLa* region, blue box) hotspots projected onto the barley QSM genetic map. The bold red and blue lines in each box represent the bins associated with *Mla1* and *MlLa*, respectively. Inset on the bottom right lists the transfer of association between hotspots. Chr, chromosome; eQTL, expression Quantitative Trait Locus; HAI, hr after inoculation; QSM, Q21861 × SM89010.

Plant transcriptomes undergo significant reprogramming in response to environmental stimuli, such as cold, salinity, drought, or pathogen stress. A marked example of this reprogramming is the transfer of association with Chr 2HL (Bins 63, 65–68) at 16 HAI, and then to Chr 1HS (Bins 3–8) at 32 HAI, where a total of 1470 genes come under alternate regulation postpenetration (Figure 5). Of these 1470 genes, 260 exhibited PacMan patterns of transcript accumulation, whereas 192 showed patterns similar to iPacMan.

**Figure 6:**
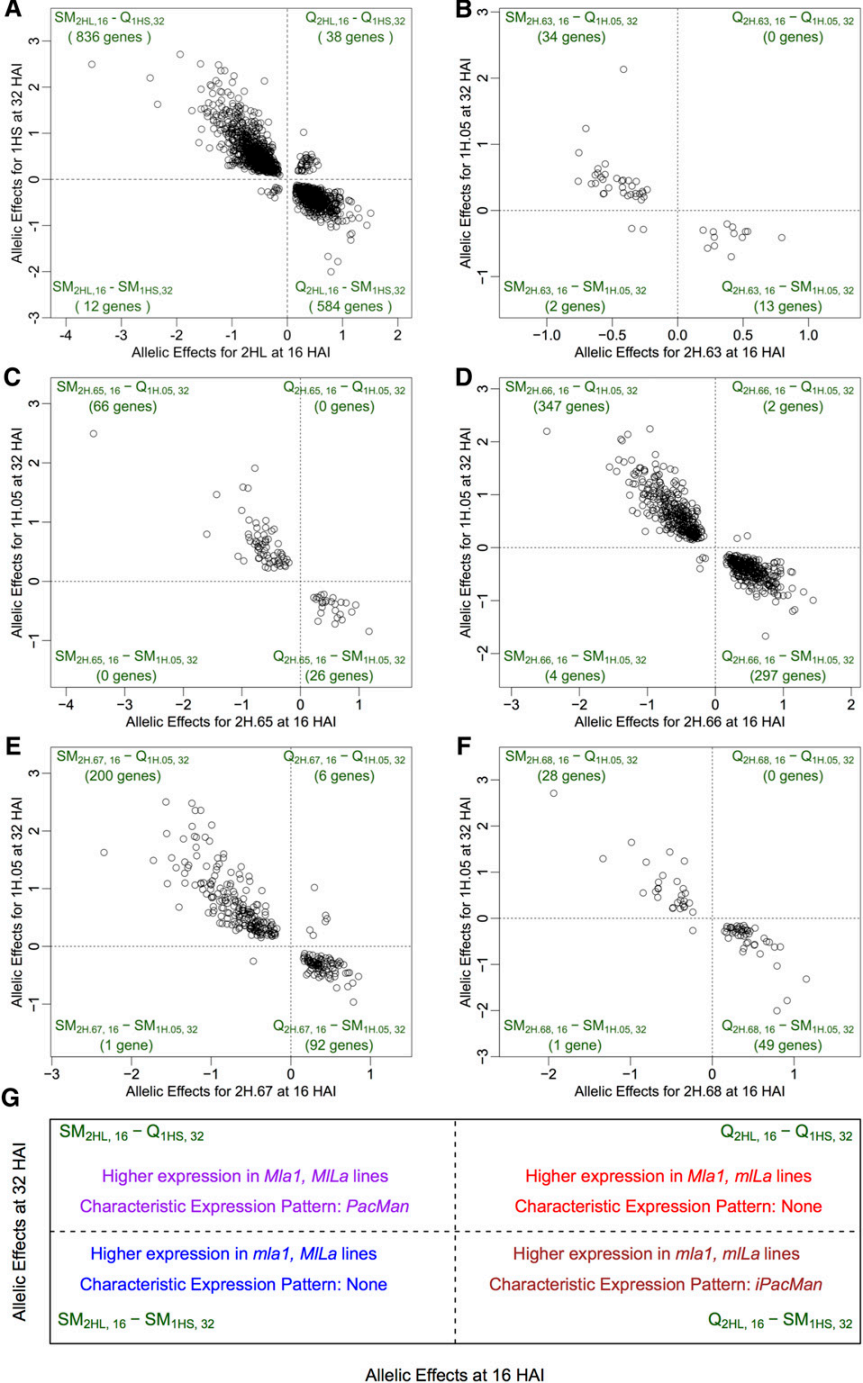
Summary of allelic effects between Chr 2HL and *Mla1* (1H.05) show modes of alternate regulation. (A) Representation of the allelic effects between 2HL at 16 HAI and 1HS at 32 HAI. The allelic effects between 1H.05 (*Mla1*) and (B) 2H.63, (C) 2H.65, (D) 2H.66, (E) 2H.67 (*MlLa*), and (F) 2H.68, are shown in respective panels. The quadrants delineated by (0,0) in each panel illustrate the four modes of regulatory transfer between hotspots and are labeled along with their number of associated genes. Chr, chromosome; HAI, hr after inoculation. Panel (G) summarizes the gene expression effects for each of the four quadrants.

Alternate transcriptional control suggests that postpenetration regulators at 1HS supplant those at 2HL. There are four modes of regulation between 2HL at 16 HAI (penetration) and 1HS at 32 HAI (haustoria), as shown in Table 2 and Table S8. We designated these SM_2HL,16_–Q_1HS,32_, SM_2HL,16_–SM_1HS,32_, Q_2HL,16_–Q_1HS,32_, and Q_2HL,16_–SM_1HS,32_, represented by the quadrants delineated at point (0,0) in Figure 6. Genes depicted in the upper-left-hand quadrant (SM_2HL,16_–Q_1HS,32_) display higher expression in recombinant lines where the SM allele is present on 2HL at 16 HAI, and in lines possessing the Q allele on 1HS at 32 HAI. This scenario also represents the presence of an active *MlLa* during penetration, in addition to a functioning *Mla1* during haustorial development. Thirty-one percent of the genes in this quadrant are associated with PacMan patterns of expression (*vs.* 0% iPacMan), and include, among other annotations, BLN1, RAB GTPase, and Syntaxin.

**Table 2:** Modes of alternate regulation between 2HL at penetration and 1HS in haustoria and associated expression patterns

Modes of Alternate Regulation	Number of Genes	Expression Pattern	
		PacMan	iPacMan
SM_2HL,16_–Q_1HS,32_	836	260	0
SM_2HL,16_–SM_1HS,32_	12	0	1
Q_2HL,16_–Q_1HS,32_	38	0	0
Q_2HL,16_–SM_1HS,32_	584	0	191

PacMan, genes downregulated during compatible interactions after 16 HAI, as compared to corresponding time points in incompatible interactions; iPacMan, genes upregulated from 16 to 32 HAI during compatible interactions, as compared to the equivalent time points in incompatible interactions.

Genes depicted in the lower-left-hand quadrant (SM_2HL,16_–SM_1HS,32_) display higher expression at 16 HAI in recombinant lines where the SM allele is present on 2HL, and at 32 HAI in lines possessing the SM allele on 1HS. This scenario is indicative of the presence of a functional *MlLa*, but is accompanied by the absence of *Mla1*. Genes depicted in the upper-right-hand quadrant (Q_2HL,16_–Q_1HS,32_) display higher expression at 16 HAI in recombinant lines where the Q allele is present on 2HL, and at 32 HAI in lines possessing the Q allele on 1HS. This scenario is indicative of the absence of a functional *MlLa*, but the presence of a functional *Mla1*. Genes in these two quadrants do not have a distinct expression pattern or associated pathway.

Lastly, genes depicted in the lower-right-hand quadrant (Q_2HL,16_–SM_1HS,32_) display higher expression at 16 HAI in recombinant lines where the Q allele is present on 2HL, and at 32 HAI in lines possessing the SM allele on 1HS. Similar to the second quadrant, this scenario is indicative of the absence of a functional *MlLa*, but also the absence of *Mla1*. Thirty-two percent of these genes are associated with transcript accumulation designated as iPacMan (*vs.* 0% PacMan). Among other annotations, these genes include nuclear transport factor 2, aquaporin PIP, and a calcium-binding EF hand family protein. Of the 1470 genes that show alternate regulation between 2HL at 16 HAI and 1HS at 32 HAI, 1420 switch the allele responsible for higher expression (SM_2HL,16_ to Q_1HS,32_ or Q_2HL,16_ to SM_1HS,32_) when association is reallocated from 2HL to 1HS.

## Discussion

We used eQTL analysis of the QSM doubled haploid barley population after infection with the fungal Ascomycete pathogen, *B. graminis* f. sp. *hordei*, to interrogate temporal control of plant immunity. Near-synchronous penetration by thousands of *Bgh* conidiospores delivers an ideal stimulus to induce dynamic reprogramming of the leaf transcriptome to response to pathogen attack. Two major clusters of *trans* eQTL hotspots on Chr 2HL and 1HS were identified along with immune response genes associated with them, corresponding to appressorial penetration and development of haustoria, respectively.

### Dynamic regulation of immunity

*Trans*-regulatory regions typically influence transcript levels via a protein intermediate and have the potential to interact with many proteins, depending on the milieu present in the cell in a given condition. A single variant may be able to activate transcription in one condition and repress it in another, resulting in a change in direction of the effect, as observed when the two carbon sources, glucose and ethanol, are compared during yeast fermentation and aerobic respiration, respectively (Smith and Kruglyak 2008). In the current eQTL study, 1470 genes associated with 2HL during penetration were repurposed by 1HS at later stages of infection, but more specifically, 299 genes reallocated control from the *MlLa* locus (2H.67) to 1H.05 (*Mla1*). Finally, an additional 138 genes were identified that change association from other non-Chr 2HL positions at 16 HAI to 1H.05 (*Mla1*) at 32 HAI. Of the total 1608 (1470 + 138) genes that undergo alternate regulation, 271 exhibited PacMan patterns of expression, which is indicative of host immune signaling that is typically suppressed by pathogen effectors (Caldo *et al.* 2004). Another 197 genes displayed a contrasting pattern of transcript accumulation designated iPacMan, which we postulate represent a scenario where the pathogen induces defense, possibly by recognition of PAMPS by pattern recognition receptors, but then coopts these genes for its own purposes (Figure 4). While we focused on transcript accumulation signified by PacMan and iPacMan patterns, undoubtedly there are additional changes influenced post-transcriptionally or post-translationally, for example by small RNAs (Liu *et al.* 2014; Xu *et al.* 2014; Fei *et al.* 2016; Zhang *et al.* 2016). Nonetheless, the population-based eQTL analysis reported here supports the conclusion that these genes are part of an immune regulon that can be activated or repressed by disparate resistance loci in response to multiple infection stages.

Alternate control of gene expression suggests that one or more regulators on 1HS supersede that exerted by eQTLs on 2HL. There are four modes of alternate transcriptional control, specifically SM_2HL,16_–Q_1HS,32_, SM_2HL,16_–SM_1HS,32_, Q_2HL,16_–Q_1HS,32_, and Q_2HL,16_–SM_1HS,32_ (Table 2). For example, SM_2HL,16_ denotes that at 16 HAI recombinant lines with the SM allele at 2HL (including *MlLa* at 2H.67) had higher gene expression compared to the lines with the Q allele at 2HL. Similarly, Q_1HS,32_ implies that at 32 HAI the lines with the Q allele at 1HS (including *Mla* at 1H.05) had higher gene expression compared to the lines with the SM allele at 1HS. As illustrated in Figure 6A, allelic effects were interchanged (Q_2HL,16_ to SM_1HS,32_ or SM_2HL,16_ to Q_1HS,32_) for 1420 of the 1470 genes that reallocated control from 2HL at penetration to 1HS during haustorial development.

One explanation for these correlated phenomena may be multiple linked polymorphisms. *Trans*-regulatory regions are much larger targets for variation than *cis*-regulatory regions (Brem *et al.* 2002; Smith and Kruglyak 2008). Multiple mutations could accumulate at loci and, depending on the condition, could compensate differentially. The mean phenotype would be stable over conditions, yet the direction of the effect within a condition could vary across the population, as observed in the phenotypic range for lines lacking a functional *Mla1*. Another example, which is observed across taxa rather than the population, is suggested by the hotspots on Chr 1HS, including those influenced by *Mla1* (1H.05). In this case, the emerging narrative is consistent with multiple polymorphisms that influence disease resistance to diverse pathogens (Seeholzer *et al.* 2010; Periyannan *et al.* 2013; Mago *et al.* 2015; Cesari *et al.* 2016).

### Membrane trafficking regulated by immune-associated loci

To further elucidate the underlying mechanisms behind these phenomena, we queried the 2HL and 1HS associations annotated to secretory pathway, vesicle transport, and signaling G-proteins. This identified 356 probe sets including several SNAREs, Rab GTPases (part of the Ras superfamily of small GTPases), tethering factors, COPs, and CCVs (Table S5). Rab GTPases regulate vesicle formation, vesicle movement, and membrane fusion (Stenmark and Olkkonen 2001), while SNARE proteins mediate fusion of vesicles with their target membrane-bound compartments (Burri and Lithgow 2004). These processes make up the route through which cell surface proteins are trafficked from the Golgi to the plasma membrane and are recycled. In plants, membrane vesicles targeted to the cell division plane fuse with one another to form the partitioning membrane, progressing from the center to the periphery of the cell. This is seen in the two types of SNARE complexes formed by KNOLLE that are jointly needed to mediate membrane fusion in cytokinesis. One of these complexes, a KNOLLE–SNAP33–VAMP721/722 trimer (El Kasmi *et al.* 2013) (Figure S1), is localized on the plasma membrane and drives membrane fusion of vesicles destined for exocytosis (Collins *et al.* 2003). PEN1 is engaged in a similar SNARE complex mediating extracellular immunity via exocytosis also with SNAP33 and VAMP721/722 (Kwon *et al.* 2008) (Figure S1). Intriguingly, the alternate regulation exhibited by VAMP721/722, first by the *MlLa* locus at penetration and then by *Mla1* during haustorial growth, could provide clues as to how its secretion apparatus is coopted for immune responses to pathogens.

Tethering factors have emerged as key regulators of membrane traffic and organellar architecture (Dubuke and Munson 2016). The restricted subcellular localization of tethering factors and their ability to interact with RABs and SNAREs suggests that tethers participate in determining the specificity of membrane fusion. An accepted model of tether function considers them molecular “bridges” that link opposing membranes before SNARE pairing. Tethers are also implicated to function as integration switches that simultaneously transmit information to coordinate distinct processes required for membrane traffic. Additionally, CCVs selectively sort cargo at the cell membrane, *trans*-Golgi network, and endosomal compartments, whereas COPs transport proteins between the Golgi complex and the rough endoplasmic reticulum. Adaptor protein complexes are vesicle coat components and appear to be involved in cargo selection and vesicle formation. AP-2 is involved in clathrin-dependent endocytosis in which cargo proteins are incorporated into vesicles surrounded by clathrin (CCVs) that are destined for fusion with the early endosome (Happel *et al.* 2004). The μ-adaptin of AP-2 (AP2M) localizes to the plasma membrane in plants for CCV formation, similar to AP-2 in animals. AP2M is involved in ETI mediated by plasma membrane-localized disease resistance proteins, possibly by mediating endocytosis of immune receptor components from the plasma membrane (Hatsugai *et al.* 2016) (Figure S2). However, it is not the coat proteins that determine the target of a transport vesicle but the SNAREs (Sanderfoot and Raikhel 1999). Together, the coat proteins and SNAREs coordinate the trafficking of cargo between various organelles of the endomembrane system.

Membrane trafficking pathways are involved in immune receptor activation, signal transduction, and execution of multiple defense responses including programmed cell death (Teh and Hofius 2014). Although mechanisms of pathogenic modulation of endocytic, secretory, and vacuolar trafficking and their roles in plant–microbe interactions need additional investigation, these pathways appear to function in the rapid responses to environmental stimuli (Inada and Ueda 2014).

### Shared components in immunity

In previous studies, we localized a *trans* eQTL hotspot to 2H.16 that associates with an enhancer for adult plant resistance to the obligate Basidiomycete fungus *Puccinia graminis tritici* race TTKSK, more commonly known as Ug99 stem rust (Moscou *et al.* 2011b). This hotspot, unlinked to the *Bgh*-responsive eQTL described here, overtakes regulatory control of 368 genes from several unlinked loci when plants are challenged with Ug99 (Moscou *et al.* 2011b). Of these 368 genes, 97 are associated with the current *Bgh*-induced 2HL at 16 HAI, 139 with 1HS at 32 HAI, and 57 undergo alternate regulation between 2HL and 1HS (Table S6), indicating substantial conservation of defense components to these two obligate biotrophic fungi.

The *Bgh*-susceptible *Arabidopsis pen2 pad4 sag101* triple mutant transformed with *Mla1* recognizes *Mla1*-incompatible isolates o*f Bgh* (Maekawa *et al.* 2012). In order to compare MLA1-dependent signaling in *Arabidopsis* and barley, the top 100 differentially expressed genes from Maekawa *et al.* (2012) were mapped onto Barley1 probe sets; 14 of these returned high-confidence alignments (Table S6). Seven of these 14 were associated with 2HL at 16 HAI, six with 1HS at 32 HAI, and four associated with alternate regulation, first by 2HL (including *MlLa*) at penetration, and then 1HS (including *Mla1*) during development of haustoria.

Only one MLA1-dependent protein from *Arabidopsis* also aligned with a probe set associated with both *MlLa* and *Mla1* in barley. This was annotated as a putative serine/threonine-protein kinase (PBL2). PBL2 contributes to PTI signaling and defense responses downstream of LRR receptor-like serine/threonine-protein kinase (FLS2) (Zhang *et al.* 2010). Ligand-activated FLS2 is internalized in a clathrin-dependent manner that converges at ARA7 (Rab GTPase Homolog F2B) endosomes facilitating the responses required for full plant immunity (Mbengue *et al.* 2016).

### Future prospects

Plant immune systems exemplify multi-tiered signaling networks comprised of biological molecules interacting in space and time. For example, we show that of the HSP90-RAR1-SGT1 chaperone complex, SGT1 is associated with 2H.67 (*MlLa* locus) at 16 HAI, whereas HSP90 is associated with 1H.05 (*Mla1*) at 32 HAI. We also show that the transcript encoding BLN1, a negative regulator of basal defense, is associated with 2H.68 at penetration, but 1H.07 during haustorial development, inferring specific control by unlinked chromosomal regions in response to different phases of pathogen infection. The regulatory mechanisms of the many examples in this report need further study, and questions also remain whether there is an epigenetic component to these phenomena. It would be intriguing to quantify methylation sites across the QSM population at different infection stages and integrate that with the current eQTL data.

One general implication of our results is that many genetic effects on most traits are likely to be detected without testing for gene–environment interactions, provided that the relevant environmental factors are known and controlled either experimentally or statistically. However, analyses that ignore temporal gene-by-environment interactions introduce strong biases regarding the types of loci that are detected. Our focus on transcript levels as quantitative traits allowed us to study a very large number of traits simultaneously and to delineate general patterns, as well as to provide detailed examples of loci that show gene-by-environment interactions, specifically infection of a plant host by an obligate biotrophic pathogen. The quantitative details would undoubtedly differ if different species, environments, and phenotypes were studied. However, some environmental differences (for example, exposure to pathogens) can have a dramatic effect on health. Our detailed studies in a model grain crop provide examples of the types of effects that may be expected in host–parasite interactions, and thereby inform practical study design.

## Supplementary Material

Supplemental material is available online at www.g3journal.org/lookup/suppl/doi:10.1534/g3.117.300125/-/DC1.
