# Supplementary figures and images for: Interchromosomal Transfer of Immune Regulation During Infection of Barley with the Powdery Mildew Pathogen
Source: G3 (Bethesda). 2017 Aug 8;7(10):3317–29. doi: 10.1534/g3.117.300125 (PMC5633382; doi:10.1534/g3.117.300125)

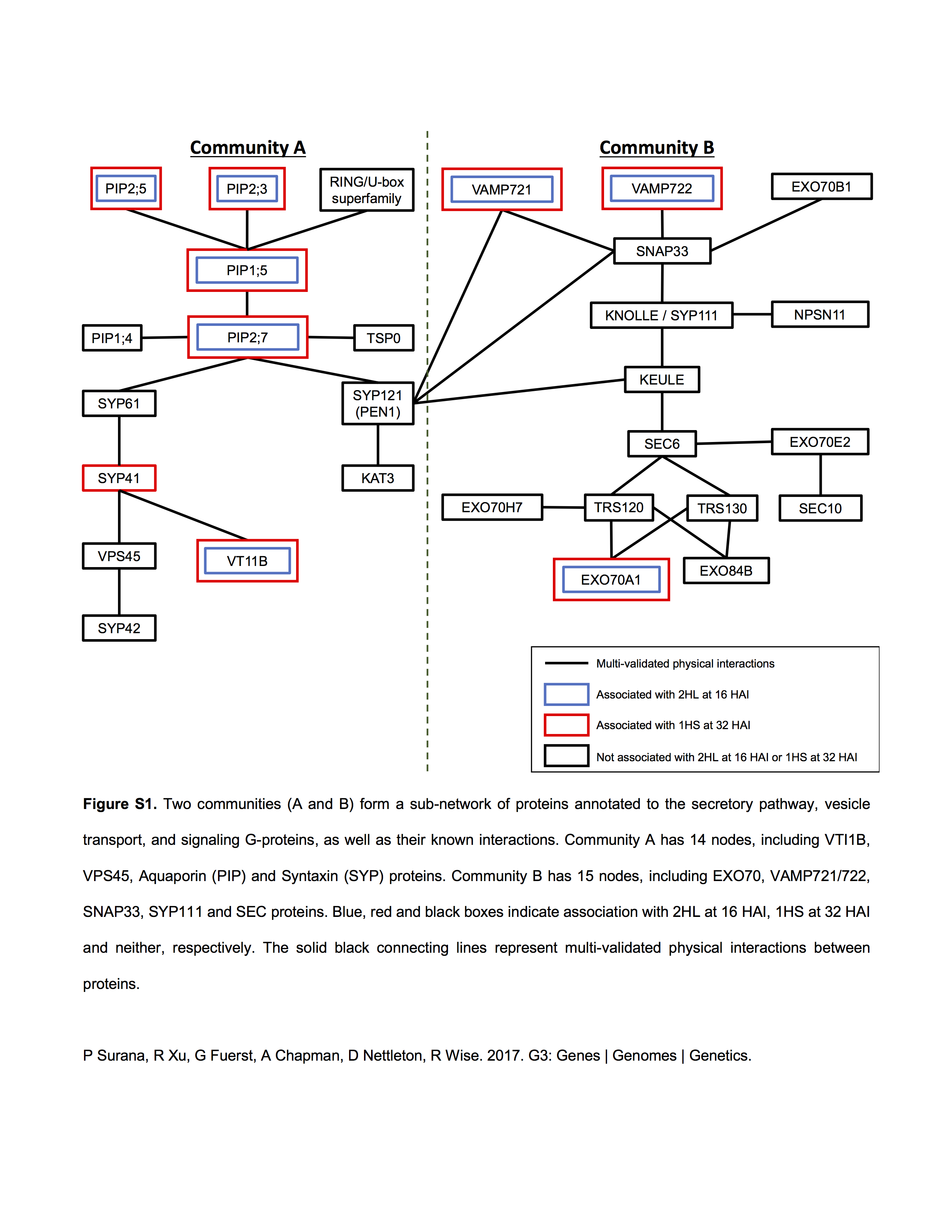

Supplement: Supplementary file 1 [file 3317FigureS1.tif]

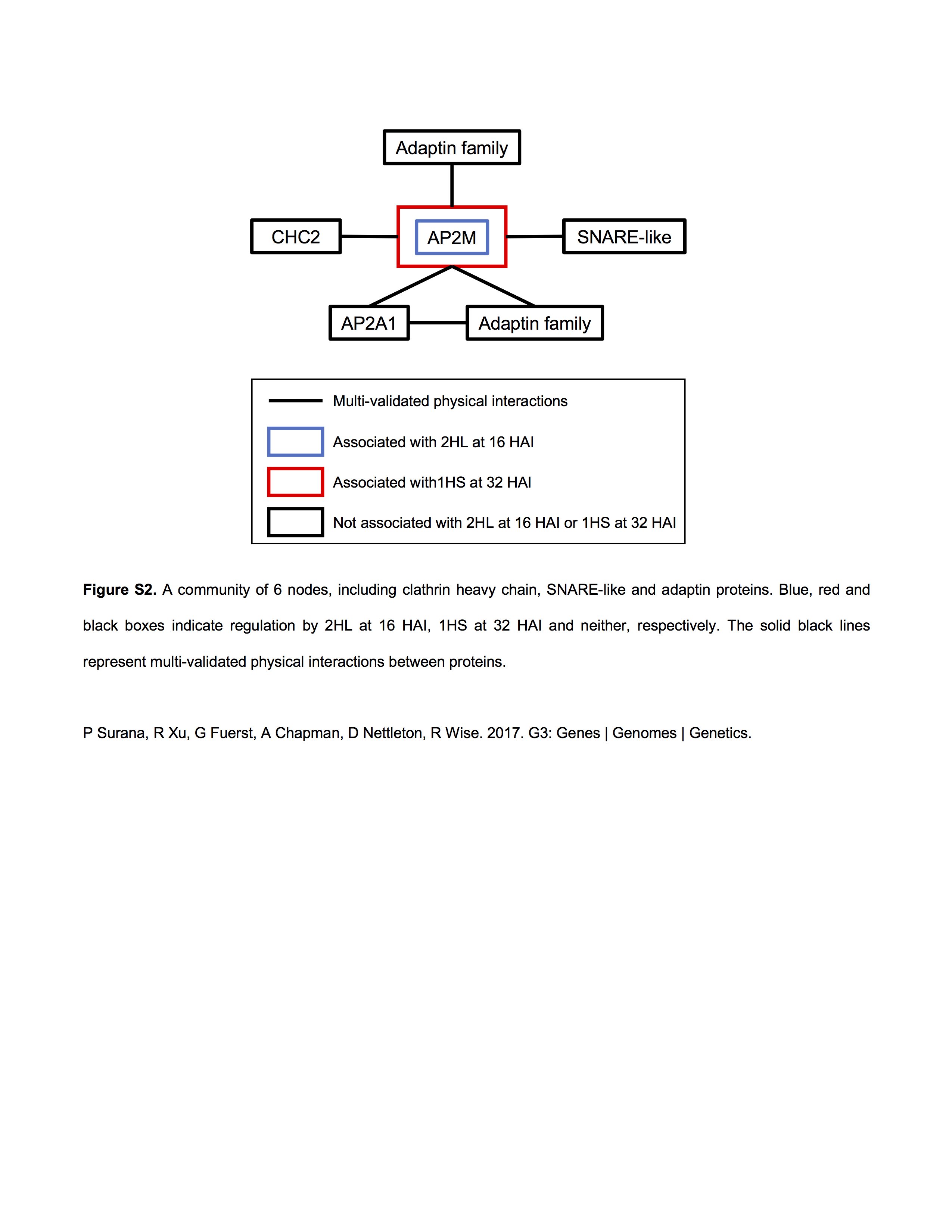

Supplement: Supplementary file 2 [file 3317FigureS2.tif]
